# Functional gastrointestinal disorders in children: agreement between Rome III and Rome IV diagnoses

**DOI:** 10.1007/s00431-021-04013-2

**Published:** 2021-03-18

**Authors:** Desiree F. Baaleman, Carlos A. Velasco-Benítez, Laura M. Méndez-Guzmán, Marc A. Benninga, Miguel Saps

**Affiliations:** 1 Emma Children’s Hospital, Amsterdam UMC, University of Amsterdam, Pediatric Gastroenterology, Amsterdam, The Netherlands; 2 Program in Clinical Medicine and Public Health, University of Granada, Granada, Spain; 3 Department of Pediatrics, Universidad del Valle, Cali, Colombia; 4 Department of Pediatrics, University of Miami, Miami, FL USA; 5 Gastroenterology and Hepatology, Amsterdam Gastroenterology Endocrinology Metabolism, Amsterdam UMC, University of Amsterdam, Meibergdreef 9, Amsterdam, Netherlands

**Keywords:** Functional gastrointestinal disorders, Prevalence, Child, Constipation, Dyspepsia

## Abstract

To evaluate the agreement between the Rome III and Rome IV criteria in diagnosing pediatric functional gastrointestinal disorders (FGIDs), we conducted a prospective cohort study in a public school in Cali, Colombia. Children and adolescents between 11 and 18 years of age were given the Spanish version of the Questionnaire on Pediatric Functional Gastrointestinal Disorders Rome III version on day 0 and Rome IV version on day 2 (48 h later). The study protocol was completed by 135 children. Thirty-nine (28.9%) children were excluded because of not following the instructions of the questionnaire. The final analysis included data of 96 children (mean 15.2 years old, SD ± 1.7, 54% girls). Less children fulfilled the criteria for an FGID according to Rome IV compared to Rome III (40.6% vs 29.2%, *p*=0.063) resulting in a minimal agreement between the two criteria in diagnosing an FGID (kappa 0.34, agreement of 70%). The prevalence of functional constipation according to Rome IV was significantly lower compared to Rome III (13.5% vs 31.3%, *p*<0.001), whereas functional dyspepsia had a higher prevalence according to Rome IV than Rome III (11.5% vs 0%).

*Conclusion*: We found an overall minimal agreement in diagnosing FGIDs according to Rome III and Rome IV criteria. This may be partly explained by the differences in diagnostic criteria. However, limitations with the use of questionnaires to measure prevalence have to be taken into account.

**Table:** 

**What is Known:** • *The Rome IV criteria replaced the previous Rome III criteria providing updated criteria to diagnose functional gastrointestinal disorders (FGIDs).* • *Differences found between Rome IV and historic Rome III FGID prevalence may have been affected by changes in prevalence over time or differences in sample characteristics.*	
**What is New:** • *We found a minimal agreement between Rome III and Rome IV FGID diagnosis, especially in the diagnoses of functional constipation, irritable bowel syndrome, and functional dyspepsia.* • *The minimal agreement may be partly explained by changes in diagnostic criteria, but limitations with the use of questionnaires to measure prevalence have to be taken into account.*

**What is Known:**

• *The Rome IV criteria replaced the previous Rome III criteria providing updated criteria to diagnose functional gastrointestinal disorders (FGIDs).*

• *Differences found between Rome IV and historic Rome III FGID prevalence may have been affected by changes in prevalence over time or differences in sample characteristics.*

**What is New:**

• *We found a minimal agreement between Rome III and Rome IV FGID diagnosis, especially in the diagnoses of functional constipation, irritable bowel syndrome, and functional dyspepsia.*

• *The minimal agreement may be partly explained by changes in diagnostic criteria, but limitations with the use of questionnaires to measure prevalence have to be taken into account.*

## Introduction

The Rome committee periodically issues criteria to facilitate the diagnosis and identification of children and adolescents with functional gastrointestinal disorders (FGIDs) [1, 2]. In 2016, the Rome criteria were adjusted based on the latest evidence. The Rome IV criteria replaced the previous Rome III criteria by renaming and changing criteria of some of the Rome III diagnoses and defining new FGIDs [3, 4]. The rationale for the changes made are described in the corresponding publications of the Rome IV criteria and in other review papers [5, 6]. To evaluate the effect of the changes made, multiple studies have compared the prevalence of FGIDs using the Rome III and Rome IV criteria [7, 8]. Those studies found significant differences in prevalence, predominantly in abdominal migraine, irritable bowel syndrome (IBS), and functional dyspepsia. However, a limitation of these studies is that all of them compared the prevalence measured at different time points (several years in between) and in different patient populations. The design of these studies does not give assurance that the differences in prevalence found are the result of changes in diagnostic criteria as opposed to changes in prevalence over time or differences in sample characteristics. Therefore, the objective of our study was to evaluate the differences and agreement between the diagnosis of an FGID according to Rome III and Rome IV criteria in one study sample and within a close timeframe. In addition, we examined the agreement between Rome III and Rome IV criteria for two major diagnostic groups, functional abdominal pain disorders and functional defecation disorders, and explored the agreement for individual FGIDs.

## Materials and methods

We conducted a prospective cohort study in October–November 2019 evaluating the agreement between Rome III and Rome IV FGIDs at a public school in Cali, Colombia. As recommended by the Rome Foundation, we used the correlating versions of self-reported pediatric questionnaires in order to diagnose children with FGIDs [9]. We used the Spanish version of the Questionnaire on Pediatric Functional Gastrointestinal Disorders, Rome III version (QPGS-III) and the Questionnaire on Pediatric Functional Gastrointestinal Disorders, Rome IV version (QPGS-IV). These questionnaires were originally published in English but were already available to us in Spanish, as we translated them for previous studies according to the guidelines of the Rome Foundation for translation and localization [7, 10, 11]. Detailed descriptions of translation processes are described in those studies. In short, two bilingual physicians reversely translated the questionnaire, after which the questionnaire was adapted by a randomly selected focus group of Columbian children. The final version was then translated back into English to assure conformity with the original English version of the questionnaire. The current study was approved by school’s teachers and principal and by the local Institutional Review Board and Human Subjects Committee.

### Inclusion and exclusion criteria

Informative material, a questionnaire covering the child’s medical history, and consent and assent forms were sent to the homes of schoolchildren/adolescents between 11 and 18 years of age. All schoolchildren 11 to 18 years of age were invited to participate in the study. Exclusion criteria were parent-reported organic gastrointestinal disorders, gastrointestinal complaints that could mimic FGIDs by causing abdominal pain (e.g., urinary tract infection), or comorbid conditions frequently associated with FGIDs (e.g., migraine headache). We did not collect any information on previous FGID complaints, diagnoses, or treatment. Moreover, after completion of both questionnaires, children who did not follow instructions on the questionnaire were excluded from analyses. Sections of the questionnaire instructed in bold letters that a specific answer to a question should prompt to skip a next section. Children who failed to follow those instructions were considered unable to complete the questionnaire accurately.

### Study protocol

Children of families who gave consent were asked to complete the QPGS-III on day 0 and the QPGS-IV on day 2 (48 h later). Before each administration, members of our research team provided instructions on the completion of the questionnaire to the children without disclosing the objective of the study. The research team stayed in the classroom until all children completed the questionnaire. Forty-eight hours after the children completed the QPGS-III, the research team distributed the QPGS-IV to the same group of children. Since both questionnaires have similar questions with sometimes just minimal differences, we chose to let the children not complete both questionnaires on the same day. We wanted to avoid the children to get tired of answering questions or falsely think that they recognize a slightly different question and answer precipitately. We chose a 48-h interval in the hope that this would stimulate them to properly read and answer every question, and for the content to the responses not to change, as most questions of the questionnaires had a 30-day reference period.

### Measurements

The prevalence of FGIDs was extracted from the answers the children gave on the QPGS-III and QPGS-IV. The prevalence of, and agreement between, similar Rome III and Rome IV diagnoses were compared between groups. The Rome IV diagnoses of functional abdominal pain-not otherwise specified replaced the Rome III diagnoses of functional abdominal pain and functional abdominal pain syndrome, and the prevalence of these disorders combined were therefore compared with the single Rome IV diagnosis.

### Statistical analysis

Statistical analyses were performed using RStudio [12]. The statistical analyses included measurements of central tendency (average, standard deviation, frequency, percentage), measurements to compare the prevalence of the Rome III and Rome IV criteria, and measurements to assess the agreement between the Rome III and Rome IV criteria. Prevalence according to both criteria was compared using the McNemar’s test for paired samples. A 2-sided *p* value < 0.05 was considered statistically significant. Agreement was assessed by calculation of the percentages of agreement and Cohen’s kappa (*κ*), including 95% confidence interval (CI). Kappa values for agreement were interpreted according to the following magnitude guidelines: 0–0.20 (none), 0.21–0.39 (minimal), 0.40–0.59 (weak), 0.60–0.79 (moderate), 0.80–0.90 (strong), and >0.90 (almost perfect) [13].

## Results

**Fig. 1:**
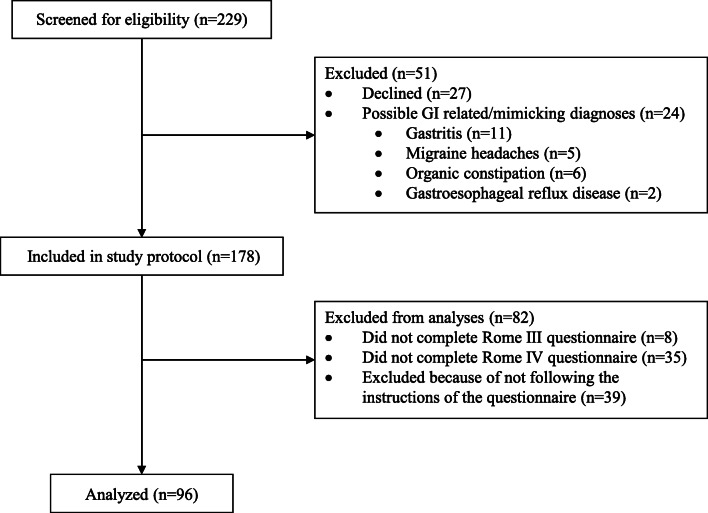
Patient flow chart

As shown in Fig. 1, we invited 229 children to participate in the study. Families of 202 (88%) children gave consent. Of those, 24 children (12%) were excluded from participation (11 gastritis, 5 migraine headaches, 6 organic constipation, and 2 gastroesophageal reflux disease). After exclusions, 178 children were included in the study protocol. Due to school absence on one of the days of the study, 43 of them (24%) did not complete one of the questionnaires. Of the remaining 135 children, 39 (29%) were excluded from analysis because of not following instructions of the questionnaire. Finally, data of 96 children was analyzed, mean age was 15.2 years (SD ± 1.7, range 11–18 years), and 54% were female.

### Prevalence of FGIDs

The prevalence of FGIDs, FGID subgroups, and individual diagnoses are shown in Table 1. Not all FGIDs were present in our study sample. The number of children meeting criteria for an FGID was higher according to Rome III compared to Rome IV criteria (40.6% vs 29.2%, respectively). However, this difference was not statistically significant (*p*=0.063). In our study sample, 7.3% of the children met criteria for multiple FGIDs according to Rome III, and 10.4% met criteria for multiple FGIDs according to Rome IV. On both testing days, functional constipation was the most common FGID. The prevalence of this disorder according to Rome IV was significantly lower (31.3% vs 13.5%, *p*<0.001). Next to this, functional dyspepsia was not prevalent according to Rome III and prevalent in 11.5% of children according to Rome IV.

**Table 1:** Prevalence of functional gastrointestinal disorders (FGIDs) according to Rome III and Rome IV criteria

	FGIDs (*n*=96)		*p* value
	Rome III (day 0)	Rome IV (day 2)	
Subjects with at least one FGIDs, *n* (%)	39 (40.6)	28 (29.2)	0.063
Subjects with multiple FGIDs, *n* (%)	7 (7.3)	10 (10.4)	
Functional nausea and vomiting disorders, *n* (%)	0 (0.0)	3 (3.1)	n/a
Functional vomiting, *n* (%)	n/a	2 (2.1)	n/a
Rumination syndrome, *n* (%)	0 (0.0)	1 (1.0)	n/a
Functional abdominal pain disorders, *n* (%)	16 (16.7)	18 (18.8)	0.813
Functional dyspepsia, *n* (%)	0 (0.0)	11 (11.5)	n/a
Postprandial distress syndrome, *n* (%)	n/a	11 (11.5)	n/a
Irritable bowel syndrome (IBS), *n* (%)	12 (12.5)	10 (10.4)	0.802
IBS-constipation, *n* (%)	n/a	3 (3.1)	n/a
IBS-mixed, *n* (%)	n/a	5 (5.2)	n/a
IBS-unclassified, *n* (%)	n/a	2 (2.1)	n/a
Abdominal migraine, *n* (%)	2 (2.1)	0 (0.0)	n/a
Functional abdominal pain-not otherwise specified (Rome IV) or functional abdominal pain and functional abdominal pain syndrome (Rome III), n (%)	2 (2.1)	2 (2.1)	1.000
Functional defecation disorders, *n* (%)	30 (31.3)	13 (13.5)	<0.001
Functional constipation, *n* (%)	30 (31.3)	13 (13.5)	<0.001

### Agreement between Rome III and Rome IV diagnoses

The agreement of diagnosis according to the Rome III and IV criteria is shown in Table 2. For diagnosing an FGID in general, the percentage of agreement was 70% with a kappa of 0.34 (95% CI, 0.16 to 0.53). None of the children who were diagnosed with the Rome III diagnoses of functional abdominal pain or functional abdominal pain syndrome were also diagnosed with the Rome IV diagnosis of functional abdominal pain-not otherwise specified.

**Table 2:** Agreement between Rome III and Rome IV functional gastrointestinal disorders (FGIDs)

	Rome IV −	Rome IV +	Agreement	Kappa (95% CI)
FGID				
Rome III –	48	9	70%	0.34 (0.16–0.53)
Rome III +	20	19		
Functional abdominal pain disorders				
Rome III –	70	10	81%	0.36 (0.12–0.59)
Rome III +	8	8		
Irritable bowel syndrome (IBS)				
Rome III –	77	7	83%	0.18 (−0.08 to 0.44)
Rome III +	9	3		
Functional Abdominal Pain-not otherwise specified				
Rome III –	92	2	96%	−0.02 (−0.04 to 0.00)
Rome III +	2	0		
Functional constipation				
Rome III –	64	2	78%	0.40 (0.20–0.59)
Rome III +	19	11		

## Discussion

The results of our study show that the agreement between Rome III and Rome IV criteria in diagnosing a child with an FGID is minimal (*κ* = 0.34; 95% CI 0.16 to 0.53). In our study sample, none of the children fulfilled Rome III criteria for functional dyspepsia, whereas 11.5% of children fulfilled Rome IV criteria for functional dyspepsia. In addition, we found that significantly less children were diagnosed with functional constipation when using the Rome IV criteria (31.3% vs 13.5%, *p*<0.001).

The minimal overall agreement in diagnosis of FGIDs may be the result of changes in diagnostic criteria between Rome III and Rome IV. A study performed in Colombia, which compared the prevalence of FGIDs according to Rome IV with a previous study using Rome III, also found a lower prevalence of FGIDs according to Rome IV [7].

In accordance with the findings of this study, we found a large difference in the prevalence of functional dyspepsia likely related to changes in diagnostic criteria [7]. In contrast with the Rome III criteria, the Rome IV criteria do not require patients to describe pain as predominant symptom and introduce two subtypes of functional dyspepsia: epigastric pain syndrome and postprandial distress syndrome. Epigastric pain syndrome is characterized by epigastric pain that is not modified with bowel movements or flatus, and postprandial distress syndrome includes children with bothersome postprandial fullness or early satiation that prevents finishing a regular meal. Based on this, children diagnosed with functional dyspepsia with Rome III would likely fulfill the criteria for epigastric pain syndrome with Rome IV [14]. As all children in our study who were diagnosed with functional dyspepsia according to Rome IV criteria had the postprandial distress syndrome subtype, it is not surprising that they would not fulfill Rome III criteria for functional dyspepsia.

However, the lower prevalence of functional constipation according to Rome IV found in the current study is unlikely caused by changes in the diagnostic criteria. As the only change made in the Rome IV criteria was the shortening of the duration of symptoms from 2 months (Rome III) to 1 month, changes in criteria should have resulted in a similar or higher number of children fulfilling the Rome IV criteria for functional constipation. In contrast to our study, an Italian study on the intra-rater agreement of Rome III and Rome IV criteria found no differences in prevalence (Rome III 22%; Rome IV 21%) and good agreement between both criteria (calculated *κ* = 0.71) [15]. In the Italian study, children who attended medical consultation or their parents completed both questionnaires consecutively, within 10 minutes, with the help of a research assistant. Difference in found agreement may be the result of differences in study samples. However, also differences in study methods may explain the better agreement found in the Italian study. First, patients with gastrointestinal complaints severe enough to consult a pediatrician may be more focused on both their symptoms and the questionnaire than children in school. Second, children completing both questionnaires consecutively are more likely to remember the answer given to the same question a few minutes before. Third, in the Italian study, a research assistant helped children and their parents in the completion of both questionnaires. Understanding whether the help of the research assistant or the parents was key in obtaining a better agreement could be instrumental for recommending the use of questionnaires in children for clinical or research purposes.

An alternative explanation for the minimal agreement found in our study could be that questionnaires may not be the best instrument to measure the presence of an FGID. The use of questionnaires to diagnose FGIDs in children on itself may result in unreliable measurements and low levels of agreement. Of the children completing all questionnaires, 39/135 (29%) did not answer the questions as instructed and were therefore excluded from the analysis; that alone questions the reliability of the questionnaire. They may have not followed instructions because of misunderstanding of the instructions, because of inappropriate reading comprehension, or because they did not pay attention to the instructions. A previous study by van Tilburg et al. studied the intra-rater reliability of FGIDs in 18 children using the QPGS-RIII [16]. Children completed the questionnaire during their outpatient pediatric gastroenterology clinic visit and again within 2 weeks at home. They found kappa values ranging between 0.22 and 0.78, though they report that given the low number of cases, these results should be considered preliminary. Moreover, they report a low agreement (kappa values ranging from −0.10 to 0.34) between child and physician diagnosis. This raises the question whether the use of questionnaires is a reliable tool to diagnose FGIDs in children. Children may just not be interested in answering questions on a questionnaire or get bored along the way and randomly answer them. Indeed, high intra-rater reliability rates (kappa values ranging from 0.86 to 0.99) of the QPGS-III are reported by Ozgenc et al. who completed the questionnaires during face-to-face interviews in 48 children within a 2-week interval [17]. However, in our study, the exclusion of questionnaires of children who did not comply with the instructions on the questionnaire should have partly reduced the possible bias caused by children who randomly answered questions. Another reason for our found low levels of agreement may be that children have a (relative) poor recall of symptoms [18, 19]. Since our population consisted of a group of apparently healthy school-going children, they may have not payed attention to their (possible) symptoms, which could result in different report of symptoms on each questionnaire.

Strengths of this study include the novelty of the study and the assessment of adequate questionnaire completion, an aspect that has not been previously reported in children completing questionnaires diagnosing FGIDs. Moreover, the results of our study are based on children’s self-completion of questionnaires and not in a medical setting, which limits bias by parents as well as selection bias. However, multiple limitations should be considered. Our study included a relatively small sample with a relative high level of attrition, of Spanish-speaking children within a specific age range (11–18 years old) located at one public school in Cali, Colombia. Therefore, these results cannot be generalized to all age groups, languages, or other geographic areas. In addition, children were not formally evaluated for an organic disease, and consequently, some of the diagnoses may be inaccurate. In addition, we were not able to compare prevalence or rates of agreement of diagnoses which were not prevalent in our study sample according to Rome III and/or Rome IV criteria (e.g., aerophagia and IBS-diarrhea). Moreover, because of the low prevalence of individual FGIDs and our small sample size, the currently reported outcomes of agreement between individual FGIDs have to be interpreted with caution and should be considered preliminary. Still, the inclusion of these data may be valuable for the conceptualization of the problem and to guide sample size calculations in future studies.

In conclusion, we found an overall minimal agreement in diagnosing FGIDs according to Rome III and Rome IV criteria. Largest differences in prevalence were seen in the diagnoses of functional constipation and functional dyspepsia. This may be partly explained by the change in diagnostic criteria. However, limitations with the use of questionnaires to measure prevalence have to be taken into account. We believe that these results imply the need to research the reliability and validity of the use of self-reported questionnaires in research on pediatric FGIDs.

## Data Availability

Data are available on request from the authors.

## Abbreviations

FGID: Functional gastrointestinal disorder
IBS: Irritable bowel syndrome
QPGS-III: Questionnaire on Pediatric Functional Gastrointestinal Disorders, Rome III version
QPGS-IV: Questionnaire on Pediatric Functional Gastrointestinal Disorders, Rome IV version

## Code availability

N/A
